# Hepatitis C Eradication Improves Oncologic and Clinical Outcomes in Patients Treated With Atezolizumab Plus Bevacizumab

**DOI:** 10.1111/liv.70362

**Published:** 2025-09-22

**Authors:** Leonardo Stella, Giuseppe Cabibbo, Ciro Celsa, Roberta Ciccia, Alba Sparacino, Fabio Piscaglia, Francesco Tovoli, Andrea Arleo, Bernardo Stefanini, Massimo Iavarone, Roberta D'Ambrosio, Lucia Cerrito, Maria Pallozzi, Francesco Santopaolo, Fabio Marra, Claudia Campani, Chiara Mazzarelli, Raffaella Viganò, Raffaella Tortora, Alessio Aghemo, Stella De Nicola, Tiziana Pressiani, Lorenza Rimassa, Sherrie Bhoori, Salvatore Corallo, Laura Maiocchi, Andrea Martini, Caterina Soldà, Francesco Paolo Russo, Antonio Gasbarrini, Francesca Romana Ponziani

**Affiliations:** ^1^ Liver Unit, CEMAD Centro Malattie del'Apparato Digerente, Medicina Interna e Gastroenterologia Fondazione Policlinico Universitario Gemelli IRCCS Rome Italy; ^2^ Policlinico Universitario «Paolo Giaccone», Azienda Ospedaliera Universitaria Palermo Italy; ^3^ Department of Surgery and Cancer Imperial College London London UK; ^4^ Department of Medical and Surgical Sciences University of Bologna Bologna Italy; ^5^ Gastroenterology and Hepatology Department Fondazione IRCCS Cà Granda Ospedale Maggiore Policlinico Milan Italy; ^6^ CRC “A. M. and A. Migliavacca” Center for Liver Disease, Department of Pathophysiology and Transplantation University of Milan Milan Italy; ^7^ Department of Experimental and Clinical Medicine, Internal Medicine and Hepatology Unit University of Florence Florence Italy; ^8^ Hepatology & Gastroenteorlogy, ASST GOM Niguarda Milan Italy; ^9^ UOC Epatologia – AORN Cardarelli Napoli Italy; ^10^ Department of Biomedical Sciences Humanitas University Milan Italy; ^11^ Division of Internal Medicine and Hepatology, Department of Gastroenterology IRCCS Humanitas Research Hospital Milan Italy; ^12^ Medical Oncology and Hematology Unit Humanitas Cancer Center, IRCCS Humanitas Research Hospital Milan Italy; ^13^ Division of Hepatology HPB Surgery and Liver Transplantation, Fondazione IRCCS Istituto Nazionale Tumori Milan Italy; ^14^ Dipartimento di Medicina Interna e Terapia Medica Università di Pavia Pavia Italy; ^15^ SC Oncologia Fondazione IRCCS Policlinico San Matteo Pavia Italy; ^16^ SC Malattie Infettive 1, Fondazione IRCCS Policlinico San Matteo Pavia Italy; ^17^ Unit of Internal Medicine and Hepatology, Department of Medicine Azienda Ospedale Università Padova Padua Italy; ^18^ Oncology Unit 1 Veneto Institute of Oncology IOV‐IRCCS Padova Italy; ^19^ Gastroenterology and Multivisceral Transplant Unit, Department of Surgery, Oncology, and Gastroenterology Padua University Hospital Padua Italy; ^20^ Dipartimento di Medicina e Chirurgia Traslazionale Università Cattolica del Sacro Cuore Rome Italy

**Keywords:** cirrhosis, DAA, HCV, hepatocellular carcinoma, immunotherapy, liver decompensation, survival

## Abstract

**Background and Aims:**

Hepatitis C virus (HCV) is a key driver of hepatocellular carcinoma (HCC). However, the impact of HCV eradication on systemic therapy remains unclear. We aimed to assess the safety and efficacy of direct‐acting antivirals (DAA) in patients treated with Atezolizumab plus Bevacizumab (AtezoBev).

**Methods:**

This retrospective multicentre study included patients with HCV‐related unresectable/advanced HCC treated with AtezoBev between 2021 and 2024. Three groups of patients were compared: Group A (*n* = 22), concurrent DAA with AtezoBev; Group B (*n* = 95), antiviral therapy before AtezoBev; and Group C (*n* = 22), active infection.

**Results:**

Group A showed the longest median overall survival (42.8 months) compared to Group B (26.8 months; *p* = 0.03) and Group C (19.7 months; *p* = 0.01). Time to progression and progression‐free survival were significantly prolonged in Group A versus Groups B and C. Moreover, Group A exhibited a higher disease control rate than the other groups. Post‐DAA decompensation rates were significantly lower in Group A (4.5%) compared to Groups B (26.3%) and C (36.4%). Treatment‐related adverse events of grade ≥ 3 were similar across groups. In the multivariate competing risk analysis with adjustment for time‐dependent variables, achieving sustained virologic response during AtezoBev showed a protective effect against liver decompensation (sHR 0.02, *p* = 0.003) or tumour progression (sHR 0.14, *p* = 0.009), and was also associated with reduced mortality (HR 0.29, *p* = 0.005).

**Conclusions:**

Achieving a SVR during AtezoBev seems to improve oncologic outcomes and reduce liver decompensation in patients with unresectable/advanced HCC. An integrated therapeutic approach can optimise systemic treatment efficacy, particularly in patients eligible for conversion strategies.

**Trial Registration:**

Protocol ID: 5890

**Summary**

- Concurrent treatment of HCV during systemic therapy is not only safe and associated with a reduced risk of liver decompensation, but also improves oncologic outcomes, including delayed tumour progression and prolonged overall survival.
- These findings support the integration of antiviral therapy into the comprehensive hepato‐oncological management of patients with HCC, particularly when downstaging or conversion strategies are considered.

## 1 Introduction

Hepatocellular carcinoma (HCC) remains one of the leading causes of cancer‐related mortality worldwide, with chronic hepatitis C virus (HCV) infection playing a significant role in both the incidence and progression of this malignancy [1]. Achieving a sustained virological response (SVR), defined as undetectable serum HCV RNA 12 or 24 weeks after the completion of antiviral therapy, has been shown to reduce liver inflammation, fibrosis, the risk of liver decompensation and HCC occurrence [2]. However, the impact of HCV treatment in patients who have already developed HCC remains less well defined.

Following the introduction of direct‐acting antivirals (DAAs), initial reports suggested an increased incidence of HCC after viral clearance [3, 4]. However, subsequent studies have largely refuted this observation, and a comprehensive meta‐analysis confirmed that HCV eradication, whether achieved with DAAs or interferon (IFN)‐based therapy, does not increase the risk of HCC [5]. More recent studies showed significant benefits in terms of overall survival (OS), liver decompensation rates and HCC recurrence reduction in patients with cirrhosis and Barcelona Clinic Liver Cancer (BCLC) stage 0/A HCC with complete oncologic response who also achieve SVR [6, 7, 8]. Additionally, a recent study demonstrated that achieving SVR through DAAs is associated with a reduced risk of liver‐related mortality and improved liver function, even in patients with HCC and advanced fibrosis or cirrhosis [9].

These findings underscore the critical role of HCV eradication in improving both liver‐related and overall clinical outcomes. However, no data are currently available on the safety and benefits of HCV eradication in patients with advanced HCC receiving systemic therapy. Concerns have been raised regarding the cost‐effectiveness of antiviral treatment in this setting, given the limited life expectancy of these patients [10]. The advent of immune checkpoint inhibitors (ICIs) has changed the therapeutic landscape of advanced HCC [11, 12, 13], not only improving survival but also increasing objective response rates (ORR), thereby enabling conversion to potentially curative treatments [14].

The aim of this study was to assess the safety of concurrent DAA therapy in patients with unresectable or advanced HCC receiving Atezolizumab plus Bevacizumab (AtezoBev) and to evaluate whether HCV eradication impacts patient survival, liver decompensation and HCC treatment outcomes.

## 2 Methods

This retrospective multicenter study included 139 patients with previous or current HCV infection and unresectable or advanced HCC, treated with AtezoBev between 2021 and 2024 in 11 Italian tertiary centres (details in Supporting Information).

Eligible patients were adults (> 18 years) with HCC not amenable to surgery or locoregional therapies and treated with AtezoBev (Atezolizumab 1200 mg plus Bevacizumab 15 mg/kg every 3 weeks) as first‐line systemic therapy until progression or unacceptable toxicity. Patients were divided into three groups: A, concurrent DAA with AtezoBev; B, antiviral therapy before AtezoBev; and C, active infection.

Data collection included comorbidities, liver function parameters (Child‐Pugh score, Model for End‐Stage Liver Disease [MELD] score and albumin‐bilirubin [ALBI] score), HCC history and HCV virological characteristics. The primary outcome was DAA safety, while secondary endpoints were OS, liver decompensation, time to decompensation (TTD), decompensation‐free survival (DeFS), time to progression (TTP), progression‐free survival (PFS), ORR and disease control rate (DCR). These outcomes were compared across three groups.

HCV eradication was defined as SVR (undetectable HCV‐RNA 12 weeks [DAA‐based regimens] or 24 weeks [IFN‐based regimens] after completion of therapy). Antiviral therapy included DAAs or IFN in Groups B and C, and DAAs only in Group A. In Group A, therapy was started within 3 months of immunotherapy; DAA duration was 12 weeks for sofosbuvir/velpatasvir (SOF/VEL) and 8 weeks for glecaprevir/pibrentasvir (GLE/PIB). Group C had active infection due to lack of response to prior therapy.

Tumour progression was assessed every 12–16 weeks using contrast‐enhanced CT or MRI according to the Response Evaluation Criteria in Solid Tumours, version 1.1 (RECIST 1.1). Liver decompensation was defined as the onset of jaundice (total bilirubin higher than 3 mg/dL), ascites, variceal haemorrhage or hepatic encephalopathy. Data on the duration of systemic therapy, reasons for discontinuation and adverse events (AEs) occurring during treatment, graded according to the National Cancer Institute Common Terminology Criteria for Adverse Events (CTCAE) v5.0 (ref), were also collected. A detailed description of the study endpoints is provided in the Supporting Information.

The study was approved by the Ethics Committee 3 of the Lazio Region (protocol ID 5890) and conducted in accordance with the principles outlined in the Declaration of Helsinki. Written consent was obtained by all subjects.

### 2.1 Statistical Analysis

Descriptive statistics were used to summarise patient characteristics. Data distribution was assessed using the Shapiro–Wilk test, with continuous variables reported as median and interquartile range (IQR), and categorical variables expressed as frequency and percentage.

Comparisons between study groups were performed using the Wilcoxon test or Kruskal–Wallis test for continuous variables, and the chi‐square test or Fisher's exact test for categorical variables, as appropriate. Bonferroni correction was applied to adjust for multiple comparisons in pairwise analyses.

Kaplan–Meier survival analysis was used to estimate OS, TTP and PFS, with intergroup differences assessed using log‐rank tests. The Cox regression model was employed for multivariate analysis, incorporating time‐dependent covariates to find predictors of mortality, progression or liver decompensation (Supporting Information). Fine and Gray [15] hazard model was used to account for competing risks, allowing for the estimation of cumulative incidence functions (CIFs) for each clinical event of interest. Gray's test was applied to compare CIFs between groups, providing a non‐parametric assessment of differences in the incidence of events while accounting for the presence of competing outcomes. Missing data were minimal and handled by complete‐case analysis; no imputation methods were applied.

All statistical analyses were conducted using R version 4.4.1. A *p* value < 0.05 was considered statistically significant.

## 3 Results

Of the 139 patients included in the study, 22 belonged to Group A, 95 to Group B and 22 to Group C. The characteristics of the study population are presented in Table 1.

**TABLE 1:** Baseline characteristics of patients in the three study groups. Categorical variables are presented as frequency (%), and continuous variables as median (IQR). Significant comparisons are highlighted in bold.

	SVR during treatment (Group A)	SVR before treatment (Group B)	Active infection (Group C)	*p*	*p*	*p*	*p*
	*N* = 22	*N* = 95	*N* = 22		A vs. B	A vs. C	B vs. C
Age (years)	63 (55–69)	69 (62–76)	65 (59–79)	**0.045**	**0.01**	0.22	0.51
Male	22 (100)	67 (70.5)	17 (77.7)	**0.01**	**0.002**	0.05	0.60
BMI (kg/m^2^)	24.3 (22.3–25.9)	24.1 (22.0–26.4)	23.1 (21.1–26.4)	0.59	0.33	0.83	0.59
ECOG PS > 0	5 (22.7)	24 (25.3)	9 (40.1)	0.28	0.80	0.20	0.14
Cirrhosis	21 (95.4)	89 (93.6)	20 (90.9)	0.82	0.75	0.55	0.64
Co‐aetiologies (%)							
HBV coinfection	2 (9.1)	8 (8.4)	2 (9.1)	0.99	0.96	1.0	0.96
HIV coinfection	2 (9.1)	2 (2.1)	1 (4.5)	0.28	0.10	0.55	0.51
MASLD	2 (9.1)	19 (20.0)	1 (4.5)	0.13	0.23	0.55	0.09
History of alcohol consumption	11 (50.0)	20 (21.1)	5 (22.7)	**0.04**	**0.02**	0.11	0.96
ALBI							
1	7 (31.8)	50 (52.6)	7 (31.8)	0.08	0.08	1.0	0.08
2	15 (68.2)	45 (47.4)	15 (68.2)				
Child Pugh							
A	19 (86.4)	83 (87.4)	20 (90.9)	0.88	0.90	0.63	0.65
B	3 (13.6)	12 (12.6)	2 (9.1)				
MELD	8 (7–8)	8 (7–9)	8 (6–10)	0.82	0.60	0.71	0.69
Portal hypertension	15 (68.2)	56 (59.0)	13 (59.1)	0.72	0.42	0.53	0.99
Varices	11 (50.0)	46 (48.4)	6 (27.3)	0.18	0.89	0.12	0.07
PVT	8 (36.4)	32 (33.7)	8 (36.4)	0.95	0.81	1.0	0.81
Ascites	3 (18.2)	13 (13.7)	1 (4.5)	0.48	0.99	0.29	0.23
Hepatic encephalopathy	2 (9.1)	4 (4.2)	0 (0)	0.33	0.31	—	—
BCLC							
B	5 (22.7)	13 (13.7)	5 (22.7)	0.41	0.33	1.0	0.33
C	17 (77.3)	82 (86.3)	17 (77.3)				
Tumour nodules (number)	5 (3–7)	4 (2–5)	4 (2–6)	0.13	**0.05**	0.32	0.46
Maximum tumour size (cm)	6.0 (4.4–7.8)	5.0 (3.1–8.0)	5.8 (3.5–7.6)	0.57	0.47	0.98	0.36
Vascular invasion	11 (50.0)	47 (49.5)	10 (45.5)	0.94	0.96	0.76	0.73
Extrahepatic spread	7 (31.2)	36 (37.9)	7 (31.8)	0.79	0.59	1.0	0.59
AFP (ng/mL)	38 (14–195)	22 (5–654)	37 (12–320)	0.61	0.44	0.97	0.45
AFP > 400 ng/mL	4 (18.2)	20 (21.1)	5 (22.7)	0.93	0.76	0.86	0.98
Surgical resection^a^	1 (4.5)	30 (31.6)	3 (13.6)	**0.01**	**0.01**	0.12	0.61
RFA^a^	1 (4.5)	38 (40.0)	3 (13.6)	**0.001**	**0.001**	0.61	**0.02**
TACE^a^	3 (13.6)	37 (38.9)	3 (13.6)	**0.01**	**0.03**	1.0	**0.03**
TARE/SBRT^a^	2 (9.1)	16 (16.8)	4 (18.2)	0.63	0.52	0.66	0.98

Abbreviations: AFP, alpha‐fetoprotein; ALBI, albumin‐bilirubin; BCLC, Barcelona clinic liver cancer; BMI, body mass index; CKD, chronic kidney disease; COPD, chronic obstructive pulmonary disease; ECOG, Eastern Cooperative Oncology Group; HBV, hepatitis B virus; HIV, human immunodeficiency virus; MASLD, metabolic associated steatotic liver disease; MELD, model for end‐stage liver disease; NSBB, non‐selective beta blockers; PPI, proton pump inhibitors; PVT, portal vein thrombosis; RFA, radiofrequency ablation; TACE, transarterial chemoembolisation; TARE/SBRT, transarterial radioembolisation/stereotactic body radiation therapy.

^a^ Previous HCC treatment.

Tumour characteristics were comparable among groups in terms of BCLC stage, number and size of nodules, vascular invasion and metastatic disease. BCLC stage C was the most frequent across all groups (*p* = 0.41). Alpha‐fetoprotein (AFP) levels did not significantly differ among groups (*p* = 0.61), either when considered as absolute values or as the prevalence of patients with AFP > 400 ng/mL.

A significantly higher proportion of patients in Group B had previously undergone locoregional treatment for HCC (67.4%), compared to Group C (40.1%, *p* = 0.02) and Group A (22.7%, *p* = 0.0002). Indeed, surgical resection, thermal ablation or transarterial chemoembolization (TACE) was performed more frequently in Group B than in the other two groups (Table 1). No significant differences were observed in the use of transarterial radioembolization (TARE) or stereotactic body radiation therapy (SBRT) across groups.

### 3.1 Virological Characteristics, DAA Treatment and Safety

Median HCV‐RNA serum levels were comparable across Group A and C while HCV genotype distribution varied, with genotype 3 being the most prevalent in Group A (Table 2).

**TABLE 2:** Virologic and treatment data of patients included in the study. Categorical variables are presented as frequency (%), and continuous variables as median (IQR). Significant comparisons are highlighted in bold.

	SVR during AtezoBev (Group A)		SVR before AtezoBev (Group B)	Active infection (Group C)	*p*	*p*	*p*	*p*
	*N* = 22		*N* = 95	*N* = 22		A vs. B	A vs. C	B vs. C
	Before DAA start	After SVR	Baseline	Baseline				
ALT (U/L)	63 (37–104)	33 (28–60)	36 (22–60)	35 (27–66)	**0.009**	**0.002**	0.06	0.36
AST (U/L)	60 (51–105)	42 (37–59)	48 (25–72)	52 (33–84)	0.12	**0.04**	0.19	0.49
GGT (U/L)	140 (91–226)	104 (39–141)	80 (46–165)	103 (76–222)	**0.03**	**0.01**	0.22	0.23
ALP (U/L)	143 (122–300)	131 (104–168)	133 (94–184)	134 (107–188)	0.46	0.24	0.34	0.71
Bilirubin (mg/dL)	1.0 (0.8–1.3)	0.8 (0.6–1.7)	1.0 (0.6–1.3)	0.9 (0.5–1.4)	0.92	0.66	0.87	0.90
INR	1.1 (1.0–1.2)	1.0 (1.0–1.2)	1.1 (1–1.2)	1.1 (1–1.2)	0.81	0.79	0.83	0.52
PLT (x10^6/L)	131 (113–238)	155 (114–202)	139 (103–212)	179 (94–230)	0.74	0.85	0.74	0.42
Albumin (mg/L)	3.7 (3.2–4.0)	3.7 (3.1–4.1)	4 (3.6–4.3)	3.7 (3.3–4.2)	0.19	0.56	0.35	0.08
HCV‐RNA (UI/mL)	923 500 (203 800–2 144 860)		—	345 000 (122 600–732 000)	0.44	—	0.44	—
HCV genotype	1a: 4 (18.2) 1b: 5 (22.7) 3: 8 (36.4) Unknown: 5 (22.7)		1a: 6 (6.3) 1b: 30 (31.6) 2: 2 (2.1) 3: 6 (6.3) 4: 1 (1.1) Unknown: 54 (56.8)	1a: 1 (4.5) 1b: 3 (13.7) Unknown: 18 (81.8)	—	—	—	—
SVR by treatment	SOF‐based: 21 (95.5) PI‐based: 1 (4.5)		SOF‐based: 43 (4.3) PI‐based: 3 (3.2) Other DAAs: 5 (5.3) IFN‐based: 5 (5.3) Unknown: 39 (41.1) +RBV: 6 (6.3)	SOF‐based: 3 (13.6) IFN‐based 3 (13.6) No treatment: 16 (72.3)	—	—	—	—
IFN‐RBV experienced patients	6 (27.3)		32 (33.7)	3 (13.6)	0.17	0.62	0.46	0.07
SVR after first treatment	19 (86.4)		92 (96.8)	—	**< 0.001**	0.08	**< 0.001**	**< 0.001**

Abbreviations: ALP, alkaline phosphatase; ALT, alanine aminotransferase; AST, aspartate aminotransferase; DAA, direct‐acting antiviral; GGT, gamma‐glutamyl transferase; GLE/PIB, glecaprevir/pibrentasvir; IFN‐RBV, interferon + ribavirin; INR, international normalised ratio; OMB/PAR/DAS, ombitasvir/paritaprevir/dasabuvir; Plts, platelets; RBV, ribavirin; SOF/DAC, sofosbuvir/daclatasvir; SOF/LED, sofosbuvir/ledipasvir; SOF/SIM, sofosbuvir/simeprevir; SOF/VEL, sofosbuvir/velpatasvir; SOF/VEL/VOX, sofosbuvir/velpatasvir/voxilaprevir.

All patients completed the planned DAA treatment course without treatment interruptions. Regarding HCV treatment regimens, SOF/VEL was the most frequently prescribed regimen in Group A (81.8%), followed by sofosbuvir/velpatasvir/voxilaprevir (SOF/VEL/VOX) (13.6%) and GLE/PIB (4.5%). In Group B, treatment regimens were more heterogeneous, with SOF/VEL (18.9%) and sofosbuvir/ledipasvir (SOF/LED) (12.6%) being the most common. In this group, the median time from SVR to AtezoBev therapy start was 65.8 (47.1–85.2) months. In Group C, only 27.2% of patients received antiviral therapy, primarily SOF/VEL (13.6%) or IFN‐based regimens (13.6%) without achieving SVR, while 72.3% were treatment‐naïve.

In Group A, 21 patients received SOF/VEL for 12 weeks, while 1 patient in Group A received GLE/PIB for 8 weeks. In Group C, three patients were treated with SOF/VEL; all of them remained viraemic after DAA treatment. The overall SVR rate was 76% (19 out of 25 patients, all from Group A). Subsequently, three additional patients from Group A were treated with SOF/VEL/VOX, increasing the SVR rate to 88% (22 out of 25, all from Group A).

At baseline laboratory tests, aminotransferase and gamma glutamyl transpeptidase (GGT) levels were significantly higher in Group A than in Group B and Group C (even if not statistically significant, Table 2). Following DAA therapy, ALT and AST levels in Group A normalised, reaching values similar to those observed in Group B, and GGT levels also declined.

No treatment‐related adverse events (TRAEs) directly attributable to DAA therapy were observed. The incidence of AEs related to AtezoBev was higher in Group A (86.4%) compared to Group B (61.1%; *p* = 0.02) and Group C (54.5%; *p* = 0.02), although the difference was not statistically significant (Table S1 and Figure S1). When adjusting for treatment duration, the exposure‐adjusted incidence rate (EAIR) of any grade TRAEs was highest in Group C (78.0 events per 100 patient‐years), followed by Group A (73.9) and Group B (59.0). The crude proportion of patients experiencing grade ≥ 3 TRAEs did not significantly differ among the groups (31.8% in Group A, 34.7% in Group B and 22.7% in Group C; *p* = 0.55). However, the EAIR for grade ≥ 3 TRAEs was 33.5 events per 100 patient‐years in Group B, 32.5 in Group C and 26.1 in Group A.

### 3.2 HCC‐Related Outcomes

**FIGURE 1:**
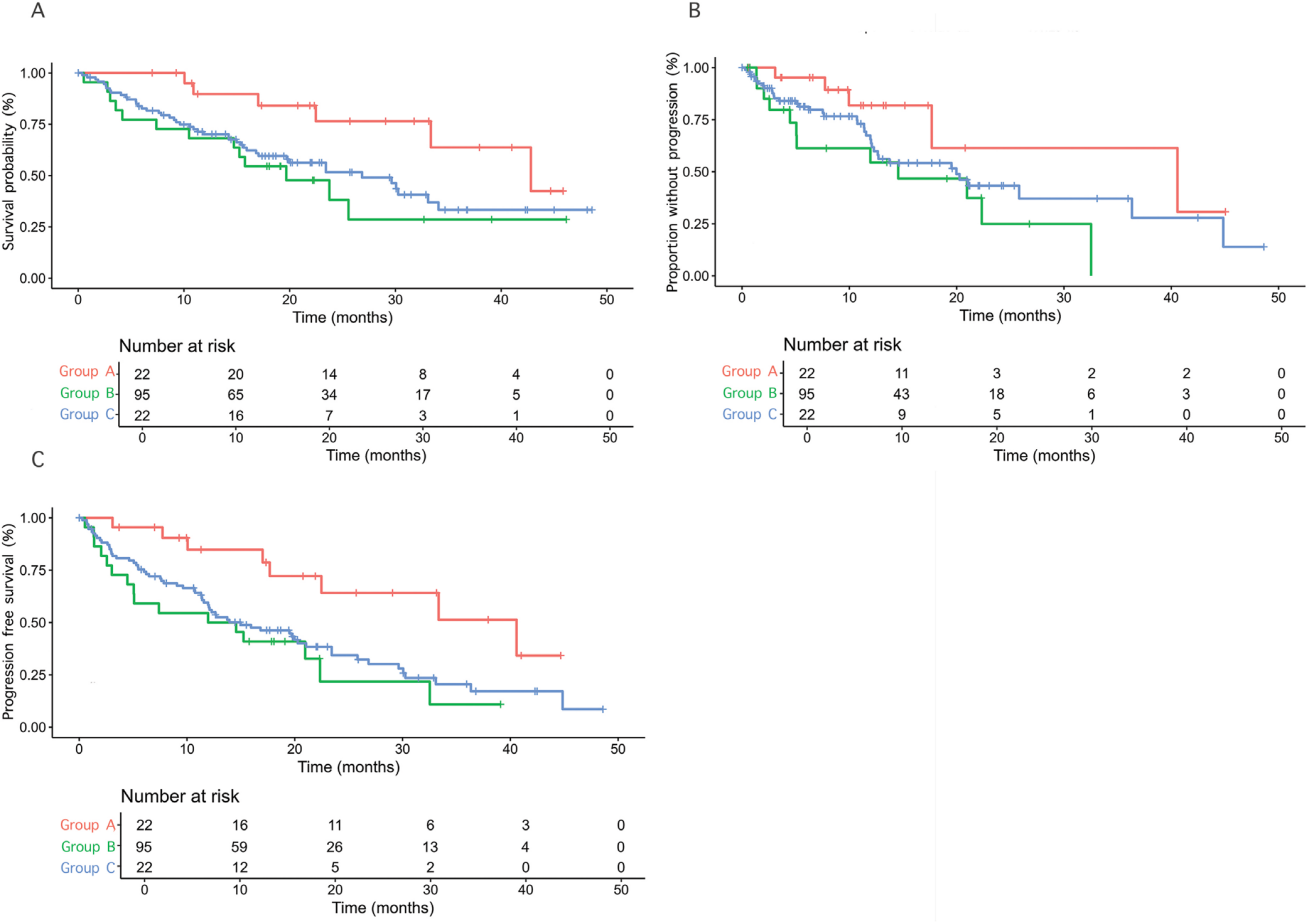
Overall survival (panel A), time to progression (panel B) and progression‐free survival (panel C) of the three study groups.

The median follow‐up was 22.4 months (14.1–33.3) for Group A, 15.6 months (8.0–23.3) for Group B and 18.0 months (8.2–22.3) for Group C, while the median duration of treatment was 10.1 months (6.8–13.6) for Group A, 7.7 months (2.9–20.7) for Group B and 5.2 months (2.6–12.1) for Group C. Mortality rates were lower in Group A (27.3%) compared to Group B (47.4%, *p* = 0.09) and significantly lower compared to Group C (59.1%, *p* = 0.03). Patients in Group A exhibited a significantly longer median OS of 42.8 months [95% CI 33.3–NA] than that observed in Group B (26.8 months [95% CI 17.1–NA]; *p* = 0.03) and Group C (19.7 months [95% CI 14.7–NA]; *p* = 0.01) (Figure 1A).

Multivariate analysis for mortality (Table S2) identified SVR achieved during AtezoBev as a protective factor (HR 0.29 [95% CI 0.12–0.69], *p* = 0.005), along with ORR (HR 0.34 [95% CI 0.18–0.66], *p* = 0.001). Conversely, ALBI grade 2–3 (HR 1.81 [95% CI 1.08–3.02], *p* = 0.02) and ECOG‐PS > 0 (HR 1.90 [95% CI 1.12–3.20], *p* = 0.002) were associated with worse survival.

The incidence of disease progression was significantly lower in Group A (22.7%) compared to Group C (54.5%, *p* = 0.03), while no significant difference emerged in comparison with Group B (40%, *p* = 0.12). Patients in Group A demonstrated a longer median TTP (40.6 months [95% CI 17.7–NA]) compared to Group C (14.6 months [95% CI 5.1–NA]; *p* = 0.02), while the difference with Group B (20 months [95% CI 12.3–NA]; *p* = 0.12) was not statistically significant (Figure 1B). PFS was also significantly longer in group A (40.6 months [95% CI 22.5–NA]) compared to Group B (15 months [95% CI 12.0–23.4]; *p* = 0.009) and Group C (13.3 months [95% CI 5.1–NA]; *p* = 0.002) (Figure 1C). In the Cox time‐dependent analysis, SVR achieved during AtezoBev was independently associated with a lower risk of disease progression (HR 0.14 [95% CI 0.05–0.72], *p* = 0.02), as well as achieving ORR at a 6 months landmark (HR 0.13 [95% CI 0.05–0.38], *p* = 0.0001; Table S2). Conversely, ECOG‐PS > 0 (HR 2.56 [95% CI 1.26–5.17], *p* = 0.01) and AFP > 400 ng/mL (HR 2.46 [95% CI 1.22–4.98], *p* = 0.01) were independent predictors of disease progression. These findings were further supported by the competing risk analysis of CIFs (Figure 3). In the overall cohort, the cumulative incidence of progression as the first event increased from 0.23 at 12 months to 0.42 at 36 months, while death without progression rose from 0.12 to 0.16 over the same period. Stratified by HCV treatment status, Group A showed the most favourable profile, with progression stabilising at 0.39 by 36 months and a moderate increase in death without prior progression (from 0.19 to 0.26). Group B showed similar progression (0.41) and competing mortality (0.16), whereas Group C exhibited the earliest and steepest progression (0.26 at 12 months to 0.49 at 36 months), with lower but increasing death without progression (0.06 to 0.10) (Figure 3B–D).

When accounting for the presence of death and liver decompensation as competing events, both SVR (subdistribution hazard ratio, sHR 0.14 [95% CI 0.03–0.61], *p* = 0.009) and ORR at 6 months (sHR 0.13 [95% CI 0.04–0.44], *p* = 0.0009) remained significantly associated with a reduced cumulative incidence of progression, further confirming their protective role (Table 3). Finally, radiological outcome was compared across groups; DCR was highest in Group A (90.1%), followed by Group B (75.8%) and Group C (59.1%), with a significant advantage for Group A over Group C (*p* = 0.01) (Table S3). Progressive disease (PD) was significantly more frequent in Group C (40.9%) compared to Group B (24.2%, *p* = 0.11) and Group A (9.1%, *p* = 0.01). No significant differences were observed in ORR among groups.

**TABLE 3:** Multivariate time‐dependent competitive risk analysis of factors predictive of decompensation, progressive disease and death. Significant comparisons are highlighted in bold.

Variable	sHR	CI low	CI high	*p*
*Death*				
ALBI grade > 1	0.86	0.15	4.93	0.87
Macrovascular portal vein invasion	0.44	0.14	1.38	0.16
Extrahepatic disease	0.63	0.18	2.21	0.47
*Progression*				
ORR	0.13	0.04	0.44	**0.0009**
SVR during treatment	0.14	0.03	0.61	**0.009**
Max nodule diameter > 5 cm	2.54	0.97	6.64	0.06
*Decompensation*				
AtezoBev trAEs ≥ 3	7.44	1.75	31.60	**0.006**
SVR during treatment	0.02	0.00	0.25	**0.003**
Child Pugh B	4.10	0.82	20.45	0.085

Abbreviations: AFP, alpha‐fetoprotein; ALBI, albumin–bilirubin grade; AtezoBev, Atezolizumab plus Bevacizumab; ECOG‐PS, Eastern Cooperative Oncology Group performance status; ORR, objective response rate; SVR, sustained virological response; trAEs, treatment‐related adverse events.

### 3.3 Liver Decompensation

During AtezoBev treatment, 42 patients (30.2%) experienced liver decompensation (25 ascites, 5 hepatic encephalopathy, 1 jaundice, 4 variceal bleeding, 7 unknown). The overall rate of decompensation from the start of AtezoBev was comparable across the groups: 38.8% in Group A, 26.3% in Group B and 36.4% in Group C (*p* = 0.3). Among decompensated patients, those classified as Child‐Pugh B (CP‐B) accounted for 4.5% in Group A, 5.3% in Group B (*p* = 0.89) and 4.5% in Group C (*p* = 1.0).

When stratifying by the timing of DAA administration and considering only decompensation events occurring after DAA treatment in Group A, a total of 34 decompensations were recorded (24.5%). In this analysis, decompensation occurred in 4.5% of patients in Group A, which was significantly lower compared to Group B (26.3% *p* = 0.03) and Group C (36.4% *p* = 0.004), with no significant difference between Group B and Group C (*p* = 0.6). No patients in Group A developed Child Pugh B status post‐DAA, compared to 5.3% in Group B (*p* = 0.58) and 0% in Group C (*p* = 1.0).

**FIGURE 2:**
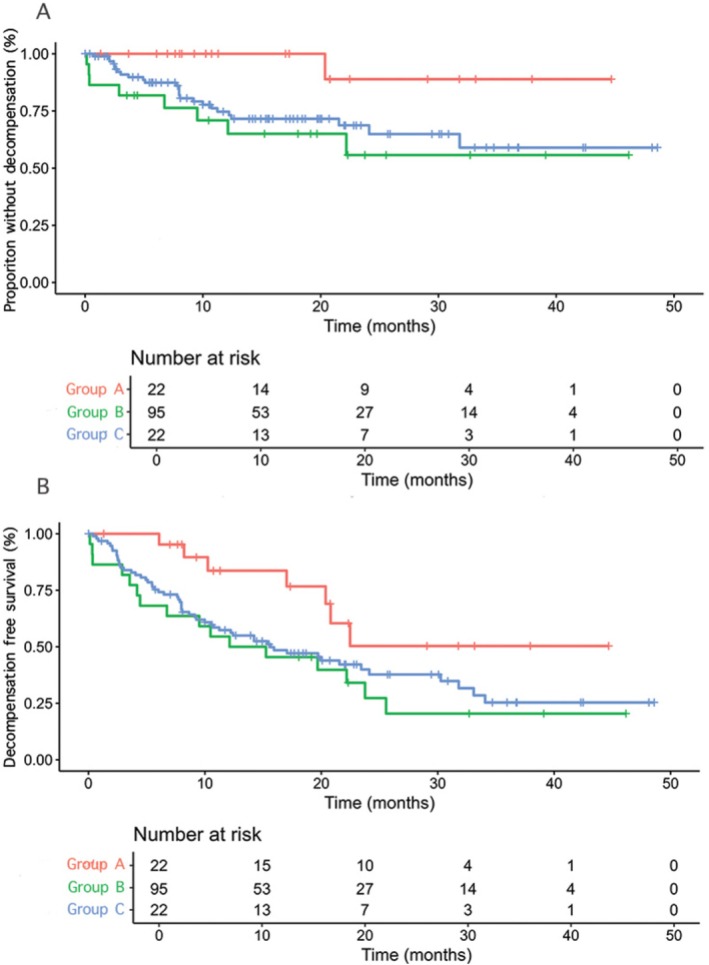
Time to decompensation (panel A) and decompensation‐free survival (panel B) of the three study groups.

The median TTD was not reached in any of the three groups (Figure 2A), with only one event observed in Group A, whereas 25% of patients in Group B experienced liver decompensation at 31.8 months [95% CI 31.8–NA] (*p* = 0.03) and 25% of those in Group C at 12.1 months [95% CI 12.1–NA] (*p* = 0.02; Figure 2A). DeFS was not reached in Group A compared to Group B (13.7 months [95% CI 6.74–NA], *p* = 0.04) and C (15.6 months [95% CI 10.7–30.3], *p* = 0.02; Figure 2B).

In the multivariate, time‐dependent, Cox regression weighted for baseline liver function and tumour burden (Table S2), the only independent predictor of post‐DAA liver decompensation was the occurrence of grade ≥ 3 TRAEs (HR 2.02 [95% CI 1.03–4.0], *p* = 0.04), while SVR during AtezoBev exerted a protective effect (HR 0.14 [95% CI 0.02–1.03], *p* = 0.05).

**FIGURE 3:**
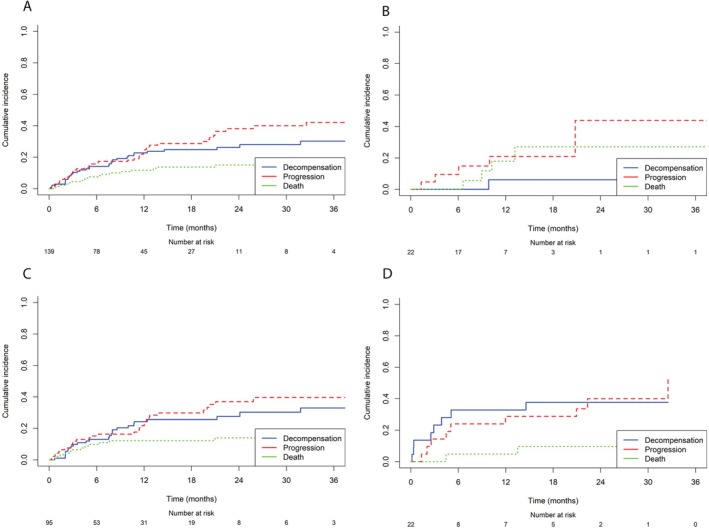
Cumulative incidence functions (CIFs) illustrating the competing risks of disease progression (dashed line), hepatic decompensation (solid line) and death without prior progression (dotted line). Panel A shows the overall cohort, while stratified analyses are reported in panel B (Group A—concurrent DAA with AtezoBev), panel C (Group B—antiviral therapy before AtezoBev) and panel D (Group C—active infection).

Using the competing risk model (Figure 3), in the overall population, the cumulative incidence of decompensation increased from 0.23 at 12 months to 0.29 at 36 months. Group A showed a consistently stable low incidence (0.03 at 12 months and 0.05 at 36 months), while decompensation rose more markedly in Group B (0.25 at 12 months and 0.32 at 36 months) and Group C (0.34 at 12 months and 0.38 at 36 months) (Figure 3B–D). Notably, decompensation in Group C increased early, with a cumulative incidence of 0.31 already at 6 months.

In the multivariate competing risk model, grade ≥ 3 TRAEs are a significant risk factor for liver decompensation (sHR 7.44 [95% CI 1.75–31.60], *p* = 0.006), whereas SVR during treatment was associated with a markedly protective effect (sHR 0.02 [95% CI 0.00–0.25], *p* = 0.003; Table 3).

## 4 Discussion

In this study, we evaluated the feasibility of DAA therapy in patients undergoing AtezoBev for unresectable or advanced HCC. Our findings indicate that DAA treatment can be safely combined with systemic therapy, and that HCV eradication significantly improves OS, TTP and PFS while reducing the risk of liver decompensation.

This is particularly relevant in unresectable HCC, where delayed tumour progression and prolonged disease control may allow conversion strategies toward curative interventions, including liver transplantation. While HCV clearance has been established as a positive prognostic factor in patients with cirrhosis [2, 16], its role in patients with HCC has been controversial.

Early studies raised concerns that DAA therapy might increase the risk of HCC recurrence [3, 4], reporting higher recurrence in BCLC 0/A HCC and increased de novo HCC incidence during or shortly after treatment [17], possibly due to immune surveillance reduction [18]. However, later analyses showed the only immunologic difference between DAA‐treated and untreated patients was downregulation of IFN genes in non‐tumour tissue, without direct evidence of increased recurrence risk [7, 19, 20, 21]. More recent studies refuted early concerns, showing that HCV eradication improves OS, particularly in early‐stage HCC (BCLC 0/A), and reduces recurrence and all‐cause mortality in patients undergoing curative resection or ablation [6].

Despite these potential benefits, many patients with advanced HCC have been excluded from DAA treatment due to safety concerns and restrictive treatment policies based on cost‐effectiveness considerations in patients with poor prognosis. Consequently, the impact of HCV eradication in advanced/unresectable HCC has remained largely unexplored. However, the introduction of ICIs has reshaped the HCC therapeutic landscape, improving prognosis and ORR, and enabling conversion strategies toward curative treatments. Given this evolving scenario, maximising liver function preservation and extending treatment duration to enhance eligibility for locoregional or surgical interventions has become a compelling clinical goal.

Our findings suggest that HCV eradication in patients undergoing AtezoBev is highly beneficial. In the competing risk analysis, group A showed the most favourable outcomes, with minimal liver decompensation and lower rates of progression; accordingly, achieving SVR during AtezoBev therapy was confirmed as a strong protective factor for progression and decompensation in the competitive risk models. However, the very low subdistribution hazard ratio observed for SVR (0.02 for decompensation) may overestimate the true magnitude of protection and should be interpreted with caution, particularly in light of the small sample size and the retrospective design of the study. The benefit observed in patients achieving SVR during immunotherapy may clearly stem from the immediate removal of an active hepatic insult, as already demonstrated [22, 23]. In contrast, those who cleared HCV prior to treatment and later developed HCC may be influenced by the residual, persistent effects of cirrhosis and other oncogenic factors (e.g., alcohol consumption, metabolic disease). This discrepancy may moreover reflect the dynamic trajectory of liver disease following HCV clearance, whereby a ‘fast improvement phase’, characterised by more pronounced hemodynamic and immunologic recovery, occurs early after viral eradication, followed by a slower, less marked phase of stabilisation. Group A may reflect this early phase, benefiting from rapid improvements in liver function and immune tone, while Group B may represent a later phase in which the early clinical benefits have stabilised. This hypothesis is supported by solid studies, demonstrating biomarker improvement and sustained, though incomplete, portal pressure decline following SVR [22, 23, 24]. Notably, as observed in HBV‐related HCC [25], patients who never received antiviral treatment experienced the poorest outcomes across all endpoints, further reinforcing the imperative of treating HCV infection even in those undergoing systemic therapy. These findings remark that the underlying aetiology of liver disease should be treated as early as possible (i.e., DAA therapy). This is especially important when systemic therapies such as ICIs are planned, as the time required to coordinate infusions or referral to oncology services should not delay antiviral therapy.

Furthermore, the impact of SVR during AtezoBev on oncological outcomes underscores the potential synergistic effect of DAAs and ICIs in modulating the tumour microenvironment (TME) and enhancing HCC treatment response [24]. Chronic HCV infection sustains immune dysfunction through persistent T‐cell exhaustion, characterised by overexpression of inhibitory molecules, such as PD‐1, LAG‐3 and TIM‐3 on CD8+ and CD4+ T cells [26]. Recent studies have shown that viral clearance with DAAs not only reduces the expression of these checkpoint molecules but also restores T‐cell effector functions, including cytotoxicity and proliferation [27, 28]. Importantly, DAA‐induced SVR leads to a reduction in the frequency of myeloid‐derived suppressor cells (MDSCs), which are known to inhibit antitumor T‐cell responses within the TME [29]. In addition to reversing immune exhaustion, SVR contributes to the normalisation of the T‐cell receptor (TCR) repertoire diversity, which may enhance the breadth and effectiveness of tumour‐specific immune surveillance. Restoration of antigen‐presenting cell (APC) functionality following HCV eradication further supports more efficient priming and activation of naïve and memory T cells, which are critical for effective responses to ICIs [30]. Furthermore, chronic HCV infection promotes a pro‐tumoral cytokine environment, characterised by elevated levels of IL‐6, TGF‐β and VEGF [31, 32]. SVR leads to a marked reduction in these cytokines, mitigating the immunosuppressive signalling pathways that can limit the efficacy of ICIs. Recent analyses of HCC treated with AtezoBev have highlighted that patients with an immune‐activated TME—characterised by greater infiltration of functional CD8+ T cells and reduced myeloid suppression—exhibit superior responses and prolonged survival compared to those with an immune‐exhausted phenotype [33]. Thus, by eradicating HCV during immunotherapy, it may be possible to shift the immune landscape toward a more favourable, immune‐activated state [34], thereby enhancing responsiveness to ICIs and improving long‐term outcomes. Evidence from immunovirological literature indicates that successful DAA therapy may partially reverse T‐cell exhaustion, as shown by reduced expression of PD‐1, TIM‐3 and TIGIT and improved proliferative capacity of HCV‐specific T cells [35]. These observations support the plausibility of our hypotheses but should be regarded as indirect and hypothesis‐generating. While limited by the small sample size, these findings nonetheless offer biological and clinical support for the concomitant use of antiviral and immunotherapeutic approaches in HCC management.

Notably, we did not observe TRAEs directly attributable to DAA throughout the study. We observed a trend toward a higher incidence of AtezoBev‐related AEs in Group A; the underlying mechanisms remain uncertain, and further studies are needed to clarify whether factors such as immune reconstitution or pharmacodynamic interactions may play a role. However, the absence of immune‐related hepatotoxicity and the similar frequency of grade ≥ 3 AtezoBev‐related AEs across groups support the feasibility of integrating DAA therapy into HCC management.

This study has several limitations. The retrospective design introduces potential selection bias. Additionally, the small sample size, particularly in Group A and Group C, may have limited the statistical power of our findings. The follow‐up duration may not have been sufficient to fully assess long‐term liver decompensation. Moreover, mechanistic data were not collected to directly support the proposed immunological synergy between DAAs and ICIs. The absence of immunologic or virologic biomarkers limits the interpretability of the observed clinical effects and precludes definitive conclusions regarding the underlying biological mechanisms, which warrant investigation in future prospective studies. Such analyses could provide mechanistic insights into the interplay between DAAs and ICIs. In addition, the prolonged median OS observed in group A may be influenced not only by the protective effect of SVR, but also by favourable baseline characteristics or selection bias (e.g., younger age, fewer prior treatments). The nearly complete survival at 36 months in this group should be interpreted with caution, as follow‐up duration and censoring may have biassed this estimate. Potential lead‐time bias further limits the interpretability of these findings. Finally, differences in access to therapy and reimbursement policies across healthcare systems may limit the generalisability of these findings and the cost‐effectiveness of extending DAAs therapy to patients with advanced HCC should be considered when applying them to diverse clinical contexts.

In conclusion, achieving SVR during AtezoBev therapy is associated with a reduced risk of both liver decompensation and tumour progression, likely through a dual mechanism involving attenuation of HCV‐related liver injury and enhanced ICI efficacy. This effect is particularly relevant for patients receiving systemic treatment with a conversion intent. If confirmed in large prospective studies, these findings could provide a strong rationale for universal HCV eradication, supporting the integration of DAA therapy into HCC management—even in patients with unresectable or advanced disease—and highlighting the need to reconsider current treatment paradigms to improve outcomes in this high‐risk population.

## Author Contributions

Leonardo Stella and Francesca Romana Ponziani developed the concept and designed the study and wrote the manuscript; Leonardo Stella, Giuseppe Cabibbo, Ciro Celsa, Roberta Ciccia, Alba Sparacino, Fabio Piscaglia, Francesco Tovoli, Andrea Arleo, Bernardo Stefanini, Massimo Iavarone, Roberta D'Ambrosio, Lucia Cerrito, Maria Pallozzi, Francesco Santopaolo, Fabio Marra, Claudia Campani, Chiara Mazzarelli, Raffaella Viganò, Raffaella Tortora, Alessio Aghemo, Stella De Nicola, Tiziana Pressiani, Lorenza Rimassa, Sherrie Bhoori, Salvatore Corallo, Laura Maiocchi, Andrea Martini, Caterina Soldà, Francesco Paolo Russo and Antonio Gasbarrini collected data; Leonardo Stella and Ciro Celsa analysed data. All authors participated to data interpretation and contributed to the revision of the final version of the manuscript.

## Ethics Statement

The study was approved by the Ethics Committee 3 of the Lazio Region.

## Consent

Written consent was obtained by all subjects.

## Conflicts of Interest

Giuseppe Cabibbo received advisory board and speaker fees for Bayer, Eisai, Ipsen, AstraZeneca, MSD, Roche, Gilead; Fabio Piscaglia received consultation fees from Astrazeneca, Bayer, Bracco, ESAOTE, EISAI, Exact Sciences, GE, IPSEN, MSD, Nerviano, Roche, Samsung, Siemens Healthineers; Francesco Tovoli received consultation fees from Roche, Ipsen, Eisai; Fabio Marra received consultation fees from Roche, MSD/EISAI, AstraZeneca, Ipsen; Massimo Iavarone participated in the advisory board and received speaker fees for Gilead Sciences, Bayer, AstraZeneca, Roche, Roche Diagnostics, EISAI, IPSEN and MSD; Roberta D'Ambrosio received advisory board and consultation fees from Gilead Science and AbbVie; Lorenza Rimassa received consulting fees from AbbVie, AstraZeneca, Basilea, Bayer, BMS, Eisai, Elevar Therapeutics, Exelixis, Genenta, Hengrui, Incyte, Ipsen, Jazz Pharmaceuticals, MSD, Nerviano Medical Sciences, Roche, Servier, Taiho Oncology, Zymeworks; lecture fees from AstraZeneca, Bayer, BMS, Eisai, Guerbet, Incyte, Ipsen, Roche, Servier; travel expenses from AstraZeneca and Servier; research grants (to Institution) from AbbVie, AstraZeneca, BeiGene, Exelixis, Fibrogen, Incyte, Ipsen, Jazz Pharmaceuticals, MSD, Nerviano Medical Sciences, Roche, Servier, Taiho Oncology, TransThera Sciences, Zymeworks; Francesca Romana Ponziani received speaker fees, advisory board fees and travel grants from Bayer, MSD, Roche, Eisai, Ipsen, Astra‐Zeneca, Gilead, Abbvie.

## Supporting information


**Table S1:** liv70362‐sup‐0001‐supinfo.docx.

## Data Availability

The data that support the findings of this study are available on request from the corresponding author. The data are not publicly available due to privacy or ethical restrictions.

## References

[1] L. Stella , F. Santopaolo , A. Gasbarrini , M. Pompili , and F. R. Ponziani , “Viral Hepatitis and Hepatocellular Carcinoma: From Molecular Pathways to the Role of Clinical Surveillance and Antiviral Treatment,” World Journal of Gastroenterology 28 (2022): 2251–2281. 35800182 10.3748/wjg.v28.i21.2251 PMC9185215

[2] F. Negro , “Residual Risk of Liver Disease After Hepatitis C Virus Eradication,” Journal of Hepatology 74 (2021): 952–963. 33276027 10.1016/j.jhep.2020.11.040

[3] M. Reig , Z. Mariño , C. Perelló , et al., “Unexpected High Rate of Early Tumor Recurrence in Patients With HCV‐Related HCC Undergoing Interferon‐Free Therapy,” Journal of Hepatology 65 (2016): 719–726. 27084592 10.1016/j.jhep.2016.04.008

[4] S. Ravi , P. Axley , D. Jones , et al., “Unusually High Rates of Hepatocellular Carcinoma After Treatment With Direct‐Acting Antiviral Therapy for Hepatitis C Related Cirrhosis,” Gastroenterology 152 (2017): 911–912. 28161225 10.1053/j.gastro.2016.12.021

[5] I. Lockart , M. G. H. Yeo , B. Hajarizadeh , G. J. Dore , and M. Danta , “HCC Incidence After Hepatitis C Cure Among Patients With Advanced Fibrosis or Cirrhosis: A Meta‐Analysis,” Hepatology 76 (2022): 139–154. 35030279 10.1002/hep.32341 PMC9303770

[6] C. Celsa , C. Stornello , P. Giuffrida , et al., “Direct‐Acting Antiviral Agents and Risk of Hepatocellular Carcinoma: Critical Appraisal of the Evidence,” Annals of Hepatology 27, no. Suppl 1 (2022): 100568. 34699987 10.1016/j.aohep.2021.100568

[7] G. Cabibbo , C. Celsa , V. Calvaruso , et al., “Direct‐Acting Antivirals After Successful Treatment of Early Hepatocellular Carcinoma Improve Survival in HCV‐Cirrhotic Patients,” Journal of Hepatology 71 (2019): 265–273. 30959157 10.1016/j.jhep.2019.03.027

[8] N. E. Rich and A. G. Singal , “Direct‐Acting Antiviral Therapy and Hepatocellular Carcinoma,” Clinical Liver Disease 17 (2021): 414–417. 34386206 10.1002/cld.1082 PMC8340318

[9] E. G. Giannini , A. Pasta , M. C. Plaz Torres , et al., “Absence of Viral Replication Is Associated With Improved Outcome in Anti‐HCV‐Positive Patients With Hepatocellular Carcinoma,” Liver International 45 (2025): e16185. 39776202 10.1111/liv.16185 PMC11707821

[10] M. Reig and G. Cabibbo , “Antiviral Therapy in the Palliative Setting of HCC (BCLC‐B and ‐C),” Journal of Hepatology 74 (2021): 1225–1233. 33582128 10.1016/j.jhep.2021.01.046

[11] R. S. Finn , S. Qin , M. Ikeda , et al., “Atezolizumab Plus Bevacizumab in Unresectable Hepatocellular Carcinoma,” New England Journal of Medicine 382 (2020): 1894–1905. 32402160 10.1056/NEJMoa1915745

[12] A.‐L. Cheng , S. Qin , M. Ikeda , et al., “Updated Efficacy and Safety Data From IMbrave150: Atezolizumab Plus Bevacizumab vs. Sorafenib for Unresectable Hepatocellular Carcinoma,” Journal of Hepatology 76 (2022): 862–873. 34902530 10.1016/j.jhep.2021.11.030

[13] A. Vogel , S. L. Chan , L. A. Dawson , et al., “Hepatocellular Carcinoma: ESMO Clinical Practice Guideline for Diagnosis, Treatment and Follow‐Up,” Annals of Oncology 36 (2025): 491–506. 39986353 10.1016/j.annonc.2025.02.006

[14] U. Cillo , E. Gringeri , F. E. D'Amico , J. Lanari , A. Furlanetto , and A. Vitale , “Hepatocellular Carcinoma: Revising the Surgical Approach in Light of the Concept of Multiparametric Therapeutic Hierarchy,” Digestive and Liver Disease 57 (2025): 809–818. 39828438 10.1016/j.dld.2024.12.003

[15] J. P. Fine and R. J. Gray , “A Proportional Hazards Model for the Subdistribution of a Competing Risk,” Journal of the American Statistical Association 94 (1999): 496–509. 10.1080/01621459.2017.1401540 PMC6502476 31073256

[16] T. Reiberger , S. Lens , G. Cabibbo , et al., “EASL Position Paper on Clinical Follow‐Up After HCV Cure,” Journal of Hepatology 81 (2024): 326–344. 38845253 10.1016/j.jhep.2024.04.007

[17] F. Conti , F. Buonfiglioli , A. Scuteri , et al., “Early Occurrence and Recurrence of Hepatocellular Carcinoma in HCV‐Related Cirrhosis Treated With Direct‐Acting Antivirals,” Journal of Hepatology 65 (2016): 727–733. 27349488 10.1016/j.jhep.2016.06.015

[18] G. Amaddeo , C. T. Nguyen , P. Maillé , et al., “Intrahepatic Immune Changes After Hepatitis c Virus Eradication by Direct‐Acting Antiviral Therapy,” Liver International 40 (2020): 74–82. 31444947 10.1111/liv.14226

[19] R. Waziry , B. Hajarizadeh , J. Grebely , et al., “Hepatocellular Carcinoma Risk Following Direct‐Acting Antiviral HCV Therapy: A Systematic Review, Meta‐Analyses, and Meta‐Regression,” Journal of Hepatology 67 (2017): 1204–1212. 28802876 10.1016/j.jhep.2017.07.025

[20] A. G. Singal , N. E. Rich , N. Mehta , et al., “Direct‐Acting Antiviral Therapy for Hepatitis C Virus Infection Is Associated With Increased Survival in Patients With a History of Hepatocellular Carcinoma,” Gastroenterology 157 (2019): 1253–1263.e2. 31374215 10.1053/j.gastro.2019.07.040 PMC6815711

[21] W. M. Kamp , C. M. Sellers , S. Stein , J. K. Lim , and H. S. Kim , “Impact of Direct Acting Antivirals on Survival in Patients With Chronic Hepatitis C and Hepatocellular Carcinoma,” Scientific Reports 9 (2019): 17081. 31745132 10.1038/s41598-019-53051-2 PMC6864088

[22] G. Cabibbo , S. Petta , M. Barbara , et al., “Hepatic Decompensation Is the Major Driver of Death in HCV‐Infected Cirrhotic Patients With Successfully Treated Early Hepatocellular Carcinoma,” Journal of Hepatology 67 (2017): 65–71, 10.1016/j.jhep.2017.01.033. 28192185

[23] C. Celsa , G. Cabibbo , C. A. M. Fulgenzi , et al., “Hepatic Decompensation Is the Major Driver of Mortality in Patients With HCC Treated With Atezolizumab Plus Bevacizumab: The Impact of Successful Antiviral Treatment,” Hepatology 81 (2024): 837–852, 10.1097/HEP.0000000000001026. 39028886

[24] K. Tajiri , H. Ito , K. Kawai , et al., “Direct‐Acting Antivirals for Hepatitis C Virus‐Infected Patients With Hepatocellular Carcinoma,” World Journal of Hepatology 14 (2022): 1190–1199. 35978673 10.4254/wjh.v14.i6.1190 PMC9258255

[25] R. Li , W. Li , Q. Yang , et al., “Low‐Level Viremia Impairs Efficacy of Immune Checkpoint Inhibitors in Unresectable Hepatocellular Carcinoma,” Liver International 45 (2025): e70066, 10.1111/liv.70066. 40078069 PMC11904444

[26] M. Llorens‐Revull , M. I. Costafreda , A. Rico , et al., “Partial Restoration of Immune Response in Hepatitis C Patients After Viral Clearance by Direct‐Acting Antiviral Therapy,” PLoS One 16 (2021): e0254243. 34242330 10.1371/journal.pone.0254243 PMC8270431

[27] M. Spaan , G. van Oord , K. Kreefft , et al., “Immunological Analysis During Interferon‐Free Therapy for Chronic Hepatitis C Virus Infection Reveals Modulation of the Natural Killer Cell Compartment,” Journal of Infectious Diseases 213 (2016): 216–223. 26223768 10.1093/infdis/jiv391

[28] P. S. Sung and E.‐C. Shin , “Immunological Mechanisms for Hepatocellular Carcinoma Risk After Direct‐Acting Antiviral Treatment of Hepatitis C Virus Infection,” Journal of Clinical Medicine 10 (2021): 221. 33435135 10.3390/jcm10020221 PMC7827927

[29] S. Li , E. Mizukoshi , K. Kawaguchi , et al., “Alterations in Hepatocellular Carcinoma‐Specific Immune Responses Following Hepatitis C Virus Elimination by Direct‐Acting Antivirals,” International Journal of Molecular Sciences 23 (2022): 11623. 36232928 10.3390/ijms231911623 PMC9570039

[30] F. Fiehn , C. Beisel , and M. Binder , “Hepatitis C Virus and Hepatocellular Carcinoma: Carcinogenesis in the Era of Direct‐Acting Antivirals,” Current Opinion in Virology 67 (2024): 101423. 38925094 10.1016/j.coviro.2024.101423

[31] T. Gupta and N. S. Jarpula , “Hepatocellular Carcinoma Immune Microenvironment and Check Point Inhibitors‐Current Status,” World Journal of Hepatology 16 (2024): 353–365. 38577535 10.4254/wjh.v16.i3.353 PMC10989304

[32] Y. Fu , X. Guo , L. Sun , et al., “Exploring the Role of the Immune Microenvironment in Hepatocellular Carcinoma: Implications for Immunotherapy and Drug Resistance,” eLife 13 (2024): e95009. 39146202 10.7554/eLife.95009 PMC11326777

[33] D. Pfister , N. G. Núñez , R. Pinyol , et al., “NASH Limits Anti‐Tumour Surveillance in Immunotherapy‐Treated HCC,” Nature 592 (2021): 450–456. 33762733 10.1038/s41586-021-03362-0 PMC8046670

[34] S. Shrivastava , E. Wilson , B. Poonia , et al., “Augmentation of Hepatitis C Virus‐Specific Immunity and Sustained Virologic Response,” Journal of Viral Hepatitis 24 (2017): 742–749. 28267900 10.1111/jvh.12702 PMC10836410

[35] S. Osuch , K. J. Metzner , and K. Caraballo Cortés , “Reversal of T Cell Exhaustion in Chronic HCV Infection,” Viruses 12 (2020): 799, 10.3390/v12080799. 32722372 PMC7472290

