# Supplementary material for: Hepatitis C Eradication Improves Oncologic and Clinical Outcomes in Patients Treated With Atezolizumab Plus Bevacizumab
Source: Liver Int. 2025 Sep 22;45(10):e70362. doi: 10.1111/liv.70362 (PMC12452217; doi:10.1111/liv.70362)
Supplement: Supplementary file 1 — Table S1: liv70362‐sup‐0001‐supinfo.docx. [file LIV-45-0-s001.docx]

TABLE OF CONTENTS

[SUPPLEMENTARY METHODS 2](#_Toc199316730)

[*Participating Study Centers* 2](#_Toc199316731)

[*Definition of the study endpoints* 2](#_Toc199316732)

[*Cox regression and Fine and Gray multivariate analysis* 2](#_Toc199316733)

[SUPPLEMENTARY TABLES 4](#_Toc199316734)

[Supplementary Table 1 4](#_Toc199316735)

[Supplementary Table 2 6](#_Toc199316736)

[Supplementary Table 3 8](#_Toc199316737)

[SUPPLEMENTARY FIGURES 9](#_Toc199316738)

[Supplementary Figure 1 9](#_Toc199316739)

# SUPPLEMENTARY METHODS

## *Participating Study Centers*

The study was conducted across multiple high-specialty medical institutions in Italy, including Fondazione Policlinico Universitario Agostino Gemelli IRCCS (Rome), Policlinico Universitario "Paolo Giaccone" (Palermo), Azienda Ospedaliera Policlinico Universitario Sant’Orsola-Malpighi (Bologna), Fondazione IRCCS Ca’ Granda Ospedale Maggiore Policlinico (Milan), Azienda Ospedaliera Universitaria Careggi IRCCS (Florence), ASST Grande Ospedale Metropolitano Niguarda (Milan), Azienda Ospedaliera di Rilievo Nazionale "A. Cardarelli" (Naples), IRCCS Humanitas Research Hospital (Rozzano–Milan), Fondazione IRCCS Istituto Nazionale dei Tumori (Milan), Fondazione IRCCS Policlinico San Matteo (Pavia), and Azienda Ospedale–Università di Padova (Padua).

## *Definition of the study endpoints*

The efficacy and safety of Atezolizumab plus Bevacizumab were evaluated using both clinical and radiological outcomes^1^:

- Overall Survival (OS), defined as the time from the first treatment dose to patient death;

- Time To Progression (TTP), defined as the time from the first treatment dose to radiological evidence of tumor progression;

- Progression-Free Survival (PFS), defined as the time from the first treatment dose to radiological evidence of tumor progression or patient death.

- Objective Response Ratio (ORR), defined as the sum of complete (CR) and partial (PR) radiological response (CR + PR);

- Disease Control Rate (DCR), which includes CR, PR, and stable disease (SD), (CR + PR + SD).

- Time To Decompensation (TTD) was defined as the time from the first treatment dose to the occurrence of any event characterized as decompensation related to liver function deterioration (hyperbilirubinemia) or clinically relevant worsening of portal hypertension (i.e. hepatic encephalopathy, ascites, variceal bleeding)^2^.

- Decompensation-Free Survival (DFS), defined as the time from the first treatment dose to the occurrence of any event characterized as decompensation related to liver function deterioration (hyperbilirubinemia) or clinically relevant worsening of portal hypertension (i.e. hepatic encephalopathy, ascites, variceal bleeding) ^2^ or patient death.

Treatment response was assessed by experienced radiologists in each center according to RECIST 1.1,^3^ based on each center's practice, through computerized tomography (CT) scans or magnetic resonance imaging (MRI) performed for periodic disease assessment.

Safety outcomes, defined as the incidence of treatment-related adverse events (trAEs) graded ≥ 3 and/or serious adverse events (sAEs) defined as any expected or unexpected adverse event, related or unrelated to treatment resulting in death, life-threatening consequences, a persistent or significant disability or incapacity, inpatient admission or prolongation of existing hospitalization.

Portal hypertension defined by the presence of portosystemic collaterals or varices detected on imaging.

## *Cox regression and Fine and Gray multivariate analysis*

A series of Cox proportional hazards regression models and Fine and Gray competing risks models were employed to identify predictors of death, radiological progression, and hepatic decompensation in patients receiving AtezoBev therapy.

For the Cox analysis, variables were initially evaluated in univariable analysis, and those reaching a threshold of statistical or clinical relevance were subsequently included in multivariable models.

For the competing risk framework, Fine and Gray regression was applied to variables of interest, particularly in the analysis of decompensation, considering progression and death as competing events.

Time-dependent covariates included ORR and DCR, achievement of SVR during AtezoBev treatment, and the occurrence of grade ≥3 TRAEs. These variables could change over the course of treatment and were therefore modeled dynamically using extended Cox regression when appropriate, or through landmark analysis in the Fine and Gray model.

Time-independent covariates included baseline patient and disease characteristics: age >75 years, presence of portal hypertension, Child-Pugh class B, ALBI grade 2–3, ECOG performance status >0, number of nodules >5, maximum nodule size >5 cm, macrovascular invasion, EHS, baseline AFP >400 ng/mL, HBV co-infection, and MASLD co-etiology. These factors were fixed at treatment initiation and assessed accordingly.

1 Llovet JM, Montal R, Villanueva A. Randomized trials and endpoints in advanced HCC: Role of PFS as a surrogate of survival. J Hepatol. 2019;70:1262-1277.

2 D'Amico G, Bernardi M, Angeli P. Towards a new definition of decompensated cirrhosis. J Hepatol 2022;76:202-207.

3 Schwartz LH, Seymour L, Litiere S et al. RECIST 1.1 - Standardisation and disease-specific adaptations: Perspectives from the RECIST Working Group. *Eur J Cancer.* 2016;**62**:138-45.

# SUPPLEMENTARY TABLES

**Supplementary Table 1:** AtezoBev-related AEs and events occurred within the study groups. Variables are reported as frequency and percentage (%). Significant comparisons are highlighted in bold.

| **Adverse events** | **SVR during AtezoBev**  **(group A)**  **N = 22** | **SVR before AtezoBev**  **(group B)**  **N = 95** | **Active infection**  **(group C)**  **N = 22** | **p-value** | **p-value**  **A vs B** | **p-value**  **A vs C** | **p-value**  **B vs C** |
| --- | --- | --- | --- | --- | --- | --- | --- |
| **TRAEs** $\boldsymbol{\geq}$ **G3** | 7 (31.8) | 33 (34.7) | 5 (22.7) | 0.55 | 0.79 | 0.50 | 0.28 |
| **All grade TRAEs** | 18 (81.8) | 58 (61.1) | 12 (54.5) | 0.12 | 0.07 | 0.05 | 0.57 |
| Atezolizumab - related | | | | | | | |
| ICI-related colitis | 1 (4.5) | 1 (1.1) | 0 (0.0) | 0.38 | 0.25 | 1.00 | 1.00 |
| ICI-related pneumonia | 1 (4.5) | 3 (3.2) | 0 (0.0) | 0.64 | 0.75 | 1.00 | 1.00 |
| ALT and AST elevation | 4 (18.2) | 6 (6.3) | 0 (0.0) | 0.06 | 0.07 | 0.11 | 0.59 |
| Adrenal insufficiency | 0 (0.0) | 1 (1.1) | 0 (0.0) | 0.79 | 1.00 | 1.00 | 1.00 |
| Cushing syndrome | 1 (4.5) | 0 (0.0) | 0 (0.0) | 0.07 | 0.19 | 1.00 | 1.00 |
| Hypothyroidism | 1 (4.5) | 12 (12.6) | 1 (4.5) | 0.34 | 0.28 | 1.00 | 0.28 |
| DM1 | 0 (0.0) | 1 (1.1) | 0 (0.0) | 0.79 | 1.00 | 1.00 | 1.00 |
| Skin rash | 1 (4.5) | 6 (6.3) | 1 (4.5) | 0.92 | 0.75 | 1.00 | 0.75 |
| Arthralgia | 3 (13.6) | 6 (6.3) | 1 (4.5) | 0.43 | 0.25 | 0.29 | 0.75 |
| Bevacizumab - related | | | | | | | |
| PLT lowering | 2 (9.1) | 3 (3.2) | 0 (0.0) | 0.25 | 0.22 | 0.49 | 1.00 |
| Arterial hypertension | 2 (9.1) | 8 (8.4) | 2 (9.1) | 0.99 | 0.92 | 1.00 | 0.92 |
| Heart failure | 1 (4.5) | 1 (1.1) | 0 (0.0) | 0.38 | 0.25 | 1.00 | 1.00 |
| Proteinuria | 2 (9.1) | 10 (10.5) | 1 (4.5) | 0.69 | 0.84 | 0.55 | 0.39 |
| PVT | 0 (0.0) | 4 (4.2) | 0 (0.0) | 0.39 | 1.00 | 1.00 | 1.00 |
| Lower Limb ulcers | 0 (0.0) | 3 (3.2) | 0 (0.0) | 0.49 | 1.00 | 1.00 | 1.00 |
| Variceal bleeding | 1 (4.5) | 4 (4.2) | 0 (0.0) | 0.61 | 0.94 | 1.00 | 1.00 |
| Other CSPH-related GI bleeding | 1 (4.5) | 2 (2.1) | 0 (0.0) | 0.58 | 0.51 | 1.00 | 1.00 |
| Not – CSPH related GI bleeding | 3 (13.6) | 3 (3.2) | 1 (4.5) | 0.13 | 0.04 | 0.29 | 0.75 |
| Other bleeding | 2 (9.1) | 2 (2.1) | 1 (4.5) | 0.28 | 0.10 | 0.55 | 0.51 |
| Mucositis | 3 (13.6) | 6 (6.3) | 1 (4.5) | 0.43 | 0.25 | 0.29 | 0.75 |
| Not drug specific | | | | | | | |
| Osteomyelitis | 0 (0.0) | 0 (0.0) | 1 (4.5) | 0.07 | 1.00 | 1.00 | 0.19 |
| Pruritus | 0 (0.0) | 4 (4.2) | 3 (13.6) | 0.10 | 1.00 | 0.23 | 0.09 |
| Fever | 2 (9.1) | 2 (2.1) | 1 (4.5) | 0.28 | 0.10 | 0.55 | 0.51 |
| Peripheral neuropathy | 0 (0.0) | 0 (0.0) | 1 (4.5) | 0.07 | 1.00 | 1.00 | 0.19 |
| Complete AV block | 0 (0.0) | 1 (1.1) | 0 (0.0) | 0.79 | 1.00 | 1.00 | 1.00 |
| Weight loss and anorexia | 2 (9.1) | 12 (12.6) | 2 (9.1) | 0.83 | 0.64 | 1.00 | 0.64 |
| Diarrhea | 1 (4.5) | 4 (4.2) | 0 (0.0) | 0.61 | 0.94 | 1.00 | 1.00 |
| Fatigue | 4 (18.2) | 28 (29.5) | 3 (13.6) | 0.22 | 0.28 | 0.68 | 0.13 |
| Events | | | | | | | |
| Death | 6 (27.3) | 45 (47.4) | 13 (59.1) | 0.09 | 0.09 | **0.03** | 0.32 |
| Progression | 5 (22.7) | 38 (40.0) | 12 (54.5) | 0.10 | 0.12 | **0.03** | 0.21 |
| Decompensation | 1 (4.5) | 25 (26.3) | 9 (36.4) | **0.02** | **0.03** | **0.004** | 0.17 |

*Abbreviations: Gastrointestinal bleeding (GI bleeding), clinically significant portal hypertension (CSPH), alanine aminotransferase (ALT), aspartate aminotransferase (AST), platelet (PLT), immune checkpoint inhibitor (ICI), atrioventricular (AV), portal vein thrombosis (PVT), type 1 diabetes mellitus (DM1), treatment-related adverse events (trAE).*

**Supplementary Table 2:** Univariate and multivariate time-dependent analyses of factors predictive of mortality, progressive disease, and liver decompensation. Significant comparisons are highlighted in bold.

| **DEATH** | | | | |
| --- | --- | --- | --- | --- |
| **RISK FACTOR** | **HR (95%CI)** | **p-value** | **HR (95%CI)** | **p-value** |
| Age>75 years | 1.49 (0.87-2.40) | 0.16 | - | - |
| Decompensation during treatment | 1.50 (0.87-2.58) | 0.21 | - | - |
| Progressive disease | 1.49 (0.91-2.40) | 0.11 | - | - |
| Portal hypertension | 1.14 (0.68-1.90) | 0.62 | - | - |
| MASLD co-etiology | 0.61 (0.28-1.33) | 0.21 | - | - |
| HBV co-infection | 1.50 (0.73-3.30) | 0.25 | - | - |
| Child Pugh B | **2.05 (1.04-4.06)** | **0.04** | - | - |
| ALBI 2-3 | **1.70 (1.03-2.80)** | **0.03** | **1.81 (1.08-3.02)** | **0.02** |
| ECOG-PS>0 | **2.08 (1.23-3.50)** | **0.006** | **1.90 (1.12-3.20)** | **0.02** |
| Nr nodules>5 | 1.20 (0.59-2.41) | 0.62 | - | - |
| Max nodule size>5 cm | 1.39 (0.83-2.33) | 0.21 | - | - |
| Macrovascular invasion | 1.47 (0.90-2.41) | 0.12 | - | - |
| Extrahepatic spread (EHS) | 0.65 (0.39-1-11) | 0.11 | - | - |
| AFP>400 ng/mL | 1.24 (0.62-2.49) | 0.54 | - | - |
| >G3 AtezoBev-related AEs | 1.32 (0.79-2.21) | 0.28 | - | - |
| ORR | **0.33 (0.18-0.63)** | **0.0007** | **0.34 (0.18-0.66)** | **0.001** |
| DCR | **0.36 (0.22-0.60)** | **0.0007** | - | **-** |
| Achieving SVR during AtezoBev treatment | **0.38 (0.16-0.89)** | **0.02** | **0.29 (0.12-0.69)** | **0.005** |
| Achieving SVR at any time of patient’s history | 0.68 (0.37-1.25) | 0.21 | - | - |
| **PROGRESSION** | | | | |
| **RISK FACTOR** | **HR (95%CI)** | **p-value** | **HR** | **p-value** |
| Age>75 years | 1.38 (0.78-2.46) | 0.27 | **-** | **-** |
| Decompensation | 0.85 (0.45-1.58) | 0.60 | **-** | **-** |
| Portal hypertension | 1.51 (0.86-2.66) | 0.15 | **-** | **-** |
| MASLD co-etiology | 0.63 (0.27-1.47) | 0.28 | **-** | **-** |
| HBV co-infection | 0.64 (0.23-1.77) | 0.39 | **-** | **-** |
| Child Pugh B | 1.24 (0.49-3.15) | 0.65 | **-** | **-** |
| ALBI>1 | 1.18 (0.69-2.01) | 0.54 | **-** | **-** |
| ECOG-PS>0 | **1.92 (1.08-3.42)** | **0.03** | **2.56 (1.26-5.17)** | **0.01** |
| Nr nodules>5 | 0.89 (0.43-1.82) | 0.74 | **-** | **-** |
| Max nodule size>5 cm | 0.75 (0.43-1.33) | 0.33 | **-** | **-** |
| Macrovascular invasion | 1.17 (0.69-1.99) | 0.56 | **-** | **-** |
| Metastases | 0.97 (0.55-1.71) | 0.92 | **-** | **-** |
| AFP>400 ng/mL | **1.85 (0.94-3.62)** | **0.07** | **2.46 (1.22-4.98)** | **0.01** |
| >G3 AtezoBev-related AEs | 0.69 (0.37-1.31) | 0.26 | **-** | **-** |
| ORR | **0.16 (0.07-0.38)** | **0.0002** | **0.13 (0.05-0.38)** | **0.0001** |
| DCR | **0.18 (0.09-0.41)** | **0.0002** | **-** | **-** |
| Achieving SVR during AtezoBev treatment | **0.44 (0.18-1.09)** | **0.07** | **0.14 (0.05-0.72)** | **0.02** |
| Achieving SVR at any time of patient’s history | 0.60 (0.31-1.15) | 0.12 | **-** | **-** |
| **DECOMPENSATION** | | | | |
| **RISK FACTOR** | **HR (95%CI)** | **p-value** | **HR** | **p-value** |
| Age>75 years | 1.57 (0.78-3.14) | 0.20 | **-** | **-** |
| Progressive disease | 0.61 (0.29-1.27) | 0.19 | **-** | **-** |
| Portal hypertension | 1.36 (0.66-2.80) | 0.40 | **-** | **-** |
| MASLD co-etiology | 0.74 (0.29-1.91) | 0.53 | **-** | **-** |
| HBV co-infection | 0.95 (0.29-3.11) | 0.94 | **-** | **-** |
| Child Pugh B | 2.16 (0.82-5.70) | 0.12 | **-** | **-** |
| ALBI>1 | 1.68 (0.84-3.37) | 0.14 | **-** | **-** |
| ECOG-PS>0 | 1.66 (0.81-3.41) | 0.17 | **-** | **-** |
| Nr nodules>5 | 1.84 (0.60-5.60) | 0.28 | **-** | **-** |
| Max nodule size>5 cm | 0.90 (0.45-1.79) | 0.76 | **-** | **-** |
| Macrovascular invasion | 1.36 (0.69-2.67) | 0.37 | **-** | **-** |
| Metastases | 0.64 (0.31-1.32) | 0.22 | **-** | **-** |
| AFP>400 ng/mL | 1.94 (0.76-4.95) | 0.17 | **-** | **-** |
| >G3 AtezoBev-related AEs | **1.97 (0.99-3.90)** | **0.05** | **2.02 (.03-4.00)** | **0.04** |
| ORR | 0.60 (0.28-1.29) | 0.19 | **-** | **-** |
| DCR | 1.51 (0.59-3.92) | 0.39 | **-** | **-** |
| Achieving SVR during AtezoBev treatment | **0.15 (0.02-1.06)** | **0.05** | **0.14 (0.02-1.03)** | **0.05** |
| Achieving SVR at any time of patient’s history | 0.60 (0.28-1.33) | 0.21 | **-** | **-** |

*^Due to collinearity, only ALBI was introduced in multivariate analysis*

*Abbreviations: MASLD: metabolic dysfunction-associated steatotic liver disease; HBV: hepatitis B virus; ALBI: Albumin-Bilirubin score; ECOG-PS: Eastern Cooperative Oncology Group Performance Status; AFP: alpha-fetoprotein; trAEs: treatment-related adverse events; ORR: objective response rate; DCR: disease control rate; SVR: sustained virologic response.*

**Supplementary Table 3*:*** Best radiological tumor response according to the Response Evaluation Criteria in Solid Tumors, version 1.1 (RECIST 1.1) achieved during treatment within the study groups.

|  | **SVR during treatment**  **(group A)** | **SVR prior to treatment**  **(group B)** | **Active infection**  **(group C)** | **P-value** | **P-value (A vs B)** | **P-value (A vs C)** | **P-value (B vs C)** |
| --- | --- | --- | --- | --- | --- | --- | --- |
| **CR** | 1 (4.5) | 8 (8.4) | 2 (9.1) | 0.81 | 0.55 | 0.92 | 0.54 |
| **PR** | 6 (27.3) | 19 (20.0) | 6 (27.3) | 0.63 | 0.45 | 1.0 | 0.45 |
| **SD** | 13 (59.1) | 45 (47.4) | 5 (20.7) | **0.045** | 0.32 | **0.01** | **0.04** |
| **PD** | 2 (9.1) | 23 (24.2) | 9 (40.9) | **0.045** | 0.12 | **0.01** | 0.11 |
| **ORR** | 7 (31.8) | 25 (28.4) | 7 (31.8) | 0.80 | 0.60 | 1.0 | 0.60 |
| **DCR** | 20 (90.1) | 72 (75.8) | 13 (59.1) | **0.045** | 0.12 | **0.01** | 0.11 |

*Abbreviations: CR: complete response, PR: partial response; SD: stable disease; PD: progressive disease; ORR: objective response rate; DCR: disease control rate.*

# SUPPLEMENTARY FIGURES

## **Supplementary Figure 1:** Exposure-Adjusted Incidence Rates (EAIR) of any grade TRAEs and TRAEs ≥ G3 across study groups.


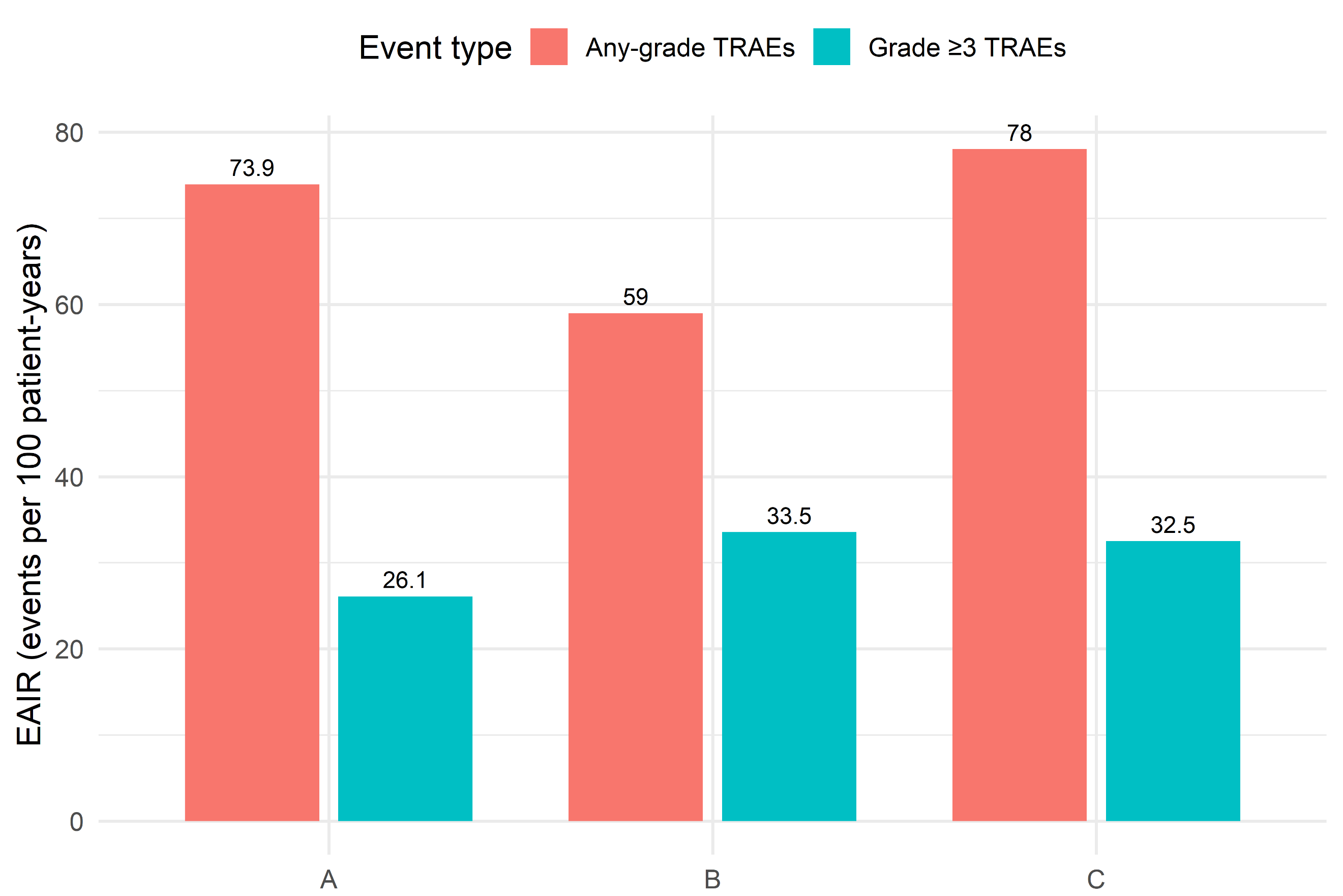


*EAIR: Exposure-Adjusted Incidence Rates; TRAEs: treatment-related adverse events*
